# Unravelling the Effect of Triacontanol in Combating Drought Stress by Improving Growth, Productivity, and Physiological Performance in Strawberry Plants

**DOI:** 10.3390/plants11151913

**Published:** 2022-07-24

**Authors:** Hossam S. El-Beltagi, Shadia A. Ismail, Nadia M. Ibrahim, Wael F. Shehata, Abdulmalik A. Alkhateeb, Hesham S. Ghazzawy, Mohamed M. El-Mogy, Eman G. Sayed

**Affiliations:** 1 Agricultural Biotechnology Department, College of Agriculture and Food Sciences, King Faisal University, Al-Ahsa 31982, Saudi Arabia; wshehata@kfu.edu.sa (W.F.S.); amalkhateeb@kfu.edu.sa (A.A.A.); 2 Biochemistry Department, Faculty of Agriculture, Cairo University, Giza 12613, Egypt; 3 Department of Potato and Vegetatively Propagated Crops, Horticulture Research Institute, Agriculture Research Center, Giza 12511, Egypt; dr.shadia134@gmail.com (S.A.I.); nadia_mohamed33@yahoo.com (N.M.I.); 4 Plant Production Department, College of Environmental Agricultural Science, El-Arish University, El-Arish 45511, Egypt; 5 Date Palm Research Center of Excellence, King Faisal University, Al-Ahsa 31982, Saudi Arabia; hghazzawy@kfu.edu.sa; 6 Central Laboratory for Date palm Research and Development, Agriculture Research Center, Giza 12511, Egypt; 7 Department of Vegetable Crops, Faculty of Agriculture, Cairo University, Giza 12613, Egypt; elmogy@agr.cu.edu.eg

**Keywords:** *Fragaria x ananassa*, quality, abiotic stress, triacontanol, antioxidant enzymes

## Abstract

To explore the effects of triacontanol (TR) on drought tolerance of strawberry plants (cv Fertona), two field experiments were carried out to study the effects of three supplementary foliar TR rates (0, 0.5, and 1 ppm) under the following three levels of water irrigation: 11 m^3^/hectare (40% of water holding capacity (WHC) severe as a drought treatment, 22 m^3^/hectare (80% of WHC) as moderate drought stress, and normal irrigation with 27 m^3^/hectare (100% of WHC) server as a control treatment. TR treatments were applied five times after 30 days from transplanting and with 15-day intervals. The results showed that drought stress (40% and 80%) markedly decreased the growth, fruit yield, and chlorophyll reading, as well as the gas exchange parameters (net photosynthetic rate, stomatal conductance, and transpiration rate). Meanwhile, drought stress at a high rate obviously increased antioxidant enzyme activities such as superoxide dismutase (SOD), peroxidase (POX), and catalase (CAT) contents in the leaves of the strawberry plants. The moderate and high drought stress rates enhanced some strawberry fruit quality parameters such as total soluble solids (TSS), vitamin C, and anthocyanin content compared to the control. Additionally, TR increased the activities of SOD, POX, and CAT. TR treatment significantly increased the chlorophyll contents, gas exchange parameters (photosynthetic rate and stomatal conductance), and water use efficiency (WUE). Plant height, fruit weight, and total biomass were increased also via TR application. Total yield per plant was increased 12.7% using 1 ppm of TR compared with the control. In conclusion, our results suggested that TR application could relieve the adverse effects of drought stress on the growth of strawberry plants by enhancing the antioxidant enzymes, photosynthesis rate, and WUE of the leaves.

## 1. Introduction

Strawberry (*Fragaria x ananassa)* is considered one of the most important vegetables belonging to the *Rosaceae* family. The fruits of strawberries contain important minerals, fibres, vitamins (especially ascorbic acid), and antioxidant compounds such as pigments (anthocyanin), phenolic compounds, and carotenoids [1,2]. Additionally, it has been suggested that daily consumption of strawberries (10–454 g) could reduce the risk of cardiovascular disease and type II diabetes [3]. According to FAOSTAT, in 2020 (https://www.fao.org/faostat/en/#data/QCL, accessed on 4 July 2022), the total world production of strawberries was 8,861,381 tonnes, which was harvested from 384,668 ha. In Egypt, in 2020, the total annual production was 597,029, which was harvested from 15,345 hectares. The Delta and northern Egypt are the regions of strawberry production in Egypt.

Drought is an environmental stress reducing plant growth, photosynthesis, and productivity of most plants [4]. It is estimated that about two-thirds of the global population will be suffering from a lack of water by the end of this century. According to UNICEF’s annual report in 2021, Egypt has an annual water deficit of approximately seven billion cubic metres, and the country may run out of water by 2025. Thus, new agricultural practices and new resistance genotypes to mitigate drought stress are immediately required. Most plant species have their own mechanisms to recover from water stress [5]. According to Flexas et al. [6], stomatal closure could be a good pointer of drought stress intensity. Stomatal conductance is generally a factor that determines the reducing of the photosynthesis rate under a water stress condition [7]. Strawberry fruits contain a high percentage of water, which means they are easily affected by a lack or excess of irrigation water [8]. Little information is available about the effect of water quantity on the quality of strawberry fruits. For example, Modise et al. [9] found negative effects of deficit irrigation on the aroma of strawberry fruits. On the other hand, proper irrigation increased yield and quality.

Triacontanol (TR) is classified as a nontoxic plant growth regulator that improves the growth and yield of plants [10]. TR is a potential phytohormone, and it is a long-chain primary fatty alcohol, CH_3_ (CH_2_)_28_ CH_2_OH. TR enhances the growth and yield of various crop species when it is foliar applied [11]. For example, foliar application of TR motivates growth in seedlings of rice [12]. Foliar application of TR increased plant height, leaf area, and biomass of hot pepper and cucumber plants [13,14]. Applying TR to the seeds or soil decreased the yield of some crops (cucumber, dry bean, carrot, tomato, barley, and radish) [15,16]. The positive role of foliar TR application on plant growth and production is related to controlling metabolic processes in plants including cell division and expansion, photosynthesis, and the activity of several enzymes [14,17]. Several previous works indicated the positive role of TR as a foliar application for reducing the harmful effects of abiotic stresses on crops such as canola [18], green gram [19], maize [20], wheat [21], common duckweed [22], seedlings of *Erythrina variegata* [23], and sweet basil [24].

Nowadays, TR is being used to improve plant tolerance to abiotic stresses such as drought, heavy metal, and salt stress [25,26]. It has been reported that exogenous TR application regulates the expression of some genes that are related to drought stress [27,28]. Additionally, TR application improves antioxidant defense systems in plants [29]. To the best of our knowledge, no previous work studied the physiological and chemical response of TR on strawberry plants under drought stress. Thus, the present work aims to evaluate the efficiency of TR in mitigating the drought stress of strawberry plants. The effect of TR on chemical composition, plant growth, and fruit quality was also studied.

## 2. Materials and Methods

### 2.1. Plant Material

This study was conducted in an experimental farm in the Faculty of Agriculture, Cairo University (located at 30°1 12″ N 31°12 5″ E), in 2020/2021 and 2021/2022. The transplants (cv. Fortuna) that were used in this experiment were cold-stored bare rooted strawberry with one crown of diameter 8–10 mm. The transplants were planted on 14 and 25 September in 2020 and 2021, respectively. The experimental unit area consisted of three rows (15 m length and 80 cm width). The distance between transplants was 30 cm. A drip irrigation system was used. The characteristics of the experimental soil was clay loam with a pH of 7.24 and EC of 0.43 ds/m. The other main elements were: HCO3.0.60 meq/L, Na^+^ 1.71 meq/L, Ca^+2^ 3.40 meq/L, Mg^+2^ 3.90 meq/L, K^+^ 0.20 meq/L, Cl^−^ 3.0 meq/L, and SO_4_^−2^ 2.30 meq/L. Calcium super phosphate (15.5% P_2_O_5_) at a rate of 108 kg hectar^−1^, ammonium sulphate (20.5% N) at rate of 144 kg hectar^−1^, and potassium sulphate (48% K_2_O) at rate of 120 kg hectar^−1^ were added in three equal parts before planting and 30 and 45 days after planting.

### 2.2. Experimental Design and Treatments

The experimental plots were arranged in a split-plot design with nine treatments. The following three levels of irrigation water in the main plot were used: 2 L/plant) 100% of water holding capacity (WHC), (1.6 L/plant) 80% of WHC, and 40% of WHC (0.8 L/plant) WHC. The following equation was used for counting the moisture content of soil mass according to Brischke and Wegener [30]: WHC% = [soilmasssaturated − soilmassovendry]soil/massovendry × 100.

**Figure 1:**
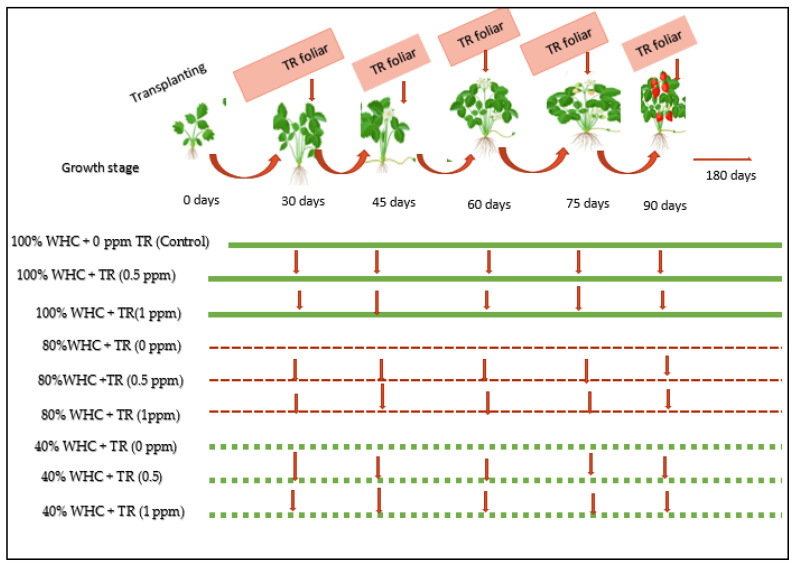
The experimental treatments and design. Different plant treatments are identified by horizontal lines as follows: 100% WHC + TR (0 ppm) control, 100% WHC + TR (0.5 ppm), 100% WHC + TR (1 ppm), 80% WHC + TR (0 ppm), 80% WHC + TR (0.5 ppm), 80% WHC + TR (1 ppm), 40% WHC + TR (0 ppm), 40% WHC + TR (0.5 ppm), and 40%WHC + TR (1 ppm).

To prepare the desire concentration of TR solution, hot distilled water with 0.1% tween 20 as a surfactant was used. TR treatments were arranged in the subplots. TR treatments were sprayed 5 times with 15-day intervals starting at the 30th day from transplanting. The complete set of treatments are summarized in Figure 1.

### 2.3. Plant Growth Parameters

Ten strawberry plants from each experimental plot were taken after 90 days from transplanting to measured plant height, leaf number, and total leaf area. Total leaf area was measured using a laser area meter CI-202 USA. Fresh samples of roots and shoots were weighed and dried in an oven at 70 °C until constant weight to measure the roots and shoots dry weights. The leaf chlorophyll reading was measured using a SPAD meter (SPAD–502, Konica Minolta Sensing, Inc., Osaka, Japan) in the fourth leaf from each treatment.

### 2.4. Gas Exchange Parameters of Strawberry Plants

Gas exchange parameters (net photosynthesis (P), transpiration rate (T), and stomata conductance (S)) were measured using an infrared gas analyser (LICOR 6400 Portable Photosynthesis System; IRGA, Licor Inc., Lincoln, NE, USA). The water use efficiency (WUE) was calculated as the P/T ratio. All measurements were made between 11:00 a.m. and 14:00 p.m. with a light intensity of 1300 mol m^−2^ s^−1^ and 80% RH. The temperature of the leaf chamber ranged from 25.2 to 27.9 °C. The volume of gas flow rate was 400 mL min^−1^. The content of CO_2_ in the air was 398 µmol mol^−1^.

### 2.5. Strawberry Fruit Yield and Its Components

The fruits were harvested from every plant at the ripe stage (¾ red colour) to measure the number of fruits per plant, the mean of fresh weight, and total yield per plant. The first four harvests were used to determine the early yield(kg·m^−2^). Total yield (t·ha^−1^) were also calculated.

### 2.6. Fruit Quality

Thirty fruits were chosen randomly from every treatment for measuring length, diameter, and firmness of fruits. Firmness was recorded using a penetrometer (FT011 Fruit Firmness Tester; Wagner Instruments, Italy) in two opposite sides of the fruits. Firmness reading values were recorded in kg/cm^2^. Total soluble solid (TSS) was measured using a hand refractometer. Titratable acidity (TA) and vitamin C was measured according to AOAC [31]. In brief, to determine TA, five fruits from each replicate were homogenised for 5 min and diluted with 50 mL distilled water and then titrated to pH 8.1 with 0.1 MNaOH. To assess vitamin C content, freshly extracted fruit (1 g) was homogenized in a mortar and pestle with metaphosphoric acid (5% metaphosphoric acid in 10% acetic acid solution in water), filtered, and treated with 85% sulphuric acid solution and 2,4-dintrophenylhydrazine before being incubated in a water bath at 60 °C for 60 min. A spectrophotometer (Genesys 10S UV-Visible) was used to measure absorbance at 520 nm to estimate the amount of vitamin C in the fruits. Total anthocyanin was determined as described previously by Doklega et al. [32]. In brief, 2 g of fruit pulp was mixed with extraction solvent (20 mL ethanol, 1.5 N HCl, 85:15) and stored at 4 °C overnight. After that, the samples were filtered into a volumetric flask. The remaining residue was washed with extraction solvent to remove the pigments and concentrated to 100 mL with extraction solvent. To calculate the anthocyanin content, the solution was measured at 535 nm absorbance. The results are presented in mg/100 g of fresh weight. The titrimetric method with 2,6-dichlorophenolindophenol was used to determine the vitamin C content in fruits [33].

### 2.7. Minerals Content in Strawberry Leaves

The samples of strawberry leaves were dried for two days in an oven dryer at 70 °C until constant weight. Then, 0.1 g of samples was digested to measured nitrogen (N), phosphor (P), and potassium (K) using sulphuric acid plus hydrogen peroxide as described previously by Sunera et al. [34]. N was measured using the Kjeldahl method as described previously by Piper [35]. P content was measured using a spectrophotometer (Shimadzu; UV-1601PC, Kyoto, Japan) according to AOAC [31]. K was determined according to Page et al. [36].

### 2.8. Proline Content and Antioxidant Enzymes of Strawberry Leaves

The free proline content was determined as described previously by Bates et al. [37]. In brief, 0.1 g of leaf samples was extracted in sulfosalicylic acid (3% 10 ML). After that, the samples were filtered using filter paper (Whatman one). Then, 2 mL of filtrated solution was added to ninhydrin and 100% glacial acetic acid (2 ML). The samples were boiled in a water bath at 100 ℃ for an hour. The process was halted by soaking the samples in ice liquid for 15–20 min, and 4 mL of toluene was added and stirred in a test-tube for 15–20 s. The samples were kept standing until the separation of toluene phase from the sample solution phase. The toluene phase was measured using a spectrophotometer (Shimadzu; UV-1601PC, Japan) with the 520 nm absorbance and proline levels expressed in µmol·g^−1^.

The ascorbate peroxidase (APX) (EC 1.11.1.11) was measured according to Nakano and Asada [38]. One unit of APX enzyme activity was defined as a decrease of 0.01 per minute in the absorbance at 290 nm. The superoxide dismutase (SOD) (EC 1.15.1.1) was determined according to Giannopolitis and Ries [39]. One unit of SOD enzyme activity was described as the amount of enzyme required to cause a 50% inhibition in the nitro blue tetrazolium chloride monohydrate (NBT) reduction. Peroxidase (POD) (EC 1.11.1.7) was analysed according to the method of Scebba et al. [40]. One unit of POD enzyme activity was regarded as an increase of 0.01 per minute in the absorbance at 470 nm. Catalase (CAT) (EC 1.11.1.6) was analysed according to Kato and Shimizu [41], and one unit of CAT enzyme activity was recognized as a decrease of 0.001 per minute in the absorbance at 240 n mm. Their specific activities were described as units mg protein. The protein concentration was determined according to Bradford [42].

### 2.9. Statistical Analysis

Data of both seasons were statistically analysed using MSTATC software. To test the significance between water irrigation levels and TR treatments, analysis of variance (ANOVA) was used using LSD at *p* < 0.05. In addition, principal component analysis (PCA) was carried out using all data points of individual response variables using origin pro 2021 version software.

## 3. Results

### 3.1. Plant Growth Parameteres

As expected, our results in Table 1, Table 2, Table 3 and Table 4 showed that both drought stress levels (80% and 40% WHC) caused marked decreases in all tested growth parameters of strawberry plants (plant height, number of leaves, fresh and dry weights of shoots and roots, SPAD chlorophyll reading, and leaf area) in both years of study compared with the well-watered condition (100% WHC).Under the well-watered condition and both drought levels, TR foliar applications at rates of 0.5 and 1 ppm significantly improved all tested growth parameters and chlorophyll readings of strawberry plants in both years of study compared with the control. The higher concentration of TR was better than the lower concentration.

### 3.2. Physiological Traits

**Figure 2:**
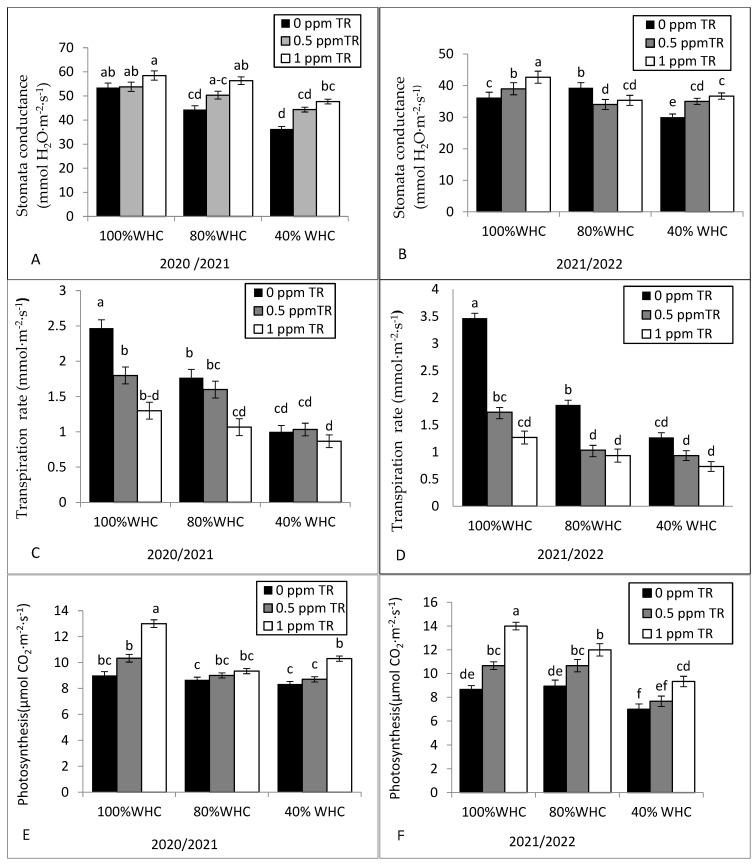

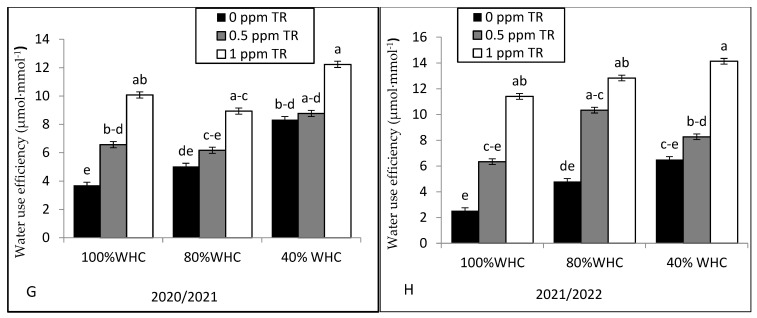
Effects of the interaction between water irrigation levels and foliar application with TR interaction on (**A**,**B**) stomata conductance in 2020 and 2021, (**C**,**D**) transpiration rate in 2020 and 2021, (**E**,**F**) photosynthesis in 2020 and 2021, and (**G**,**H**) water use efficiency in 2020 and 2021. Vertical bars represent standard errors of the mean; in each bar, values followed by different letters differ significantly at *p* = 0.05 according to the LSD test.

Figure 2 shows the impact of the interaction between water irrigation levels and TR treatments on stomatal conductance, photosynthesis, transpiration rate, and water use efficiency (WUE) in both seasons. Stomatal conductance, transpiration rate, and photosynthesis decreased under drought stress condition (Figure 2A–F). However, water use efficiency (WUE) was higher in severe stress (40% WHC) than in the well-watered condition (Figure 1G,H). Foliar application with TR improved stomatal conductance, photosynthesis, and water use efficiency, while significant decreases were observed in the transpiration rate compared to the control. Foliar application with the high TR rate (1 ppm) under well-watered irrigation (100% WHC) showed a higher photosynthesis rate in both seasons compared with the low level and the control plants.

### 3.3. Yield and Its Components

**Figure 3:**
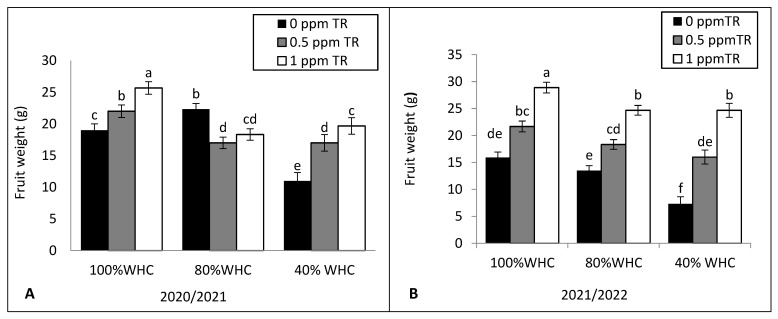

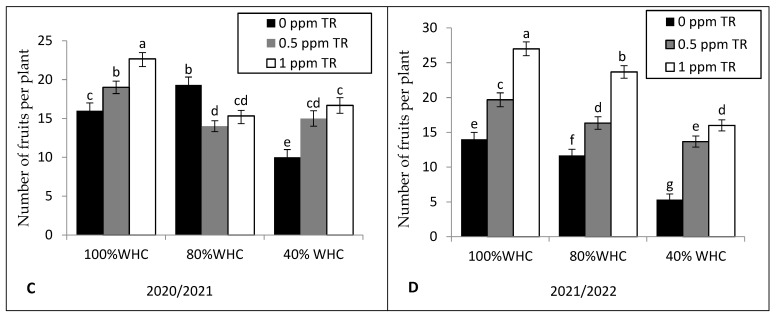
Effects of the interaction between water irrigation levels and foliar application with TR interaction on (**A**,**B**) fruit weight in 2020 and 2021 and (**C**,**D**) number of fruits per plant in 2020 and 2021. Vertical bars represent standard errors of the mean; in each bar, values followed by different letters differ significantly at *p* = 0.05 according to the LSD test.

**Figure 4:**
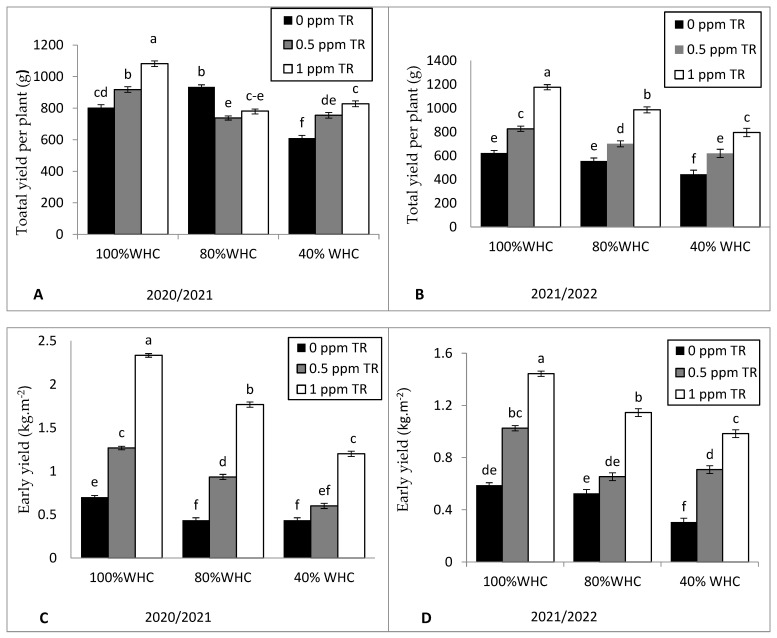

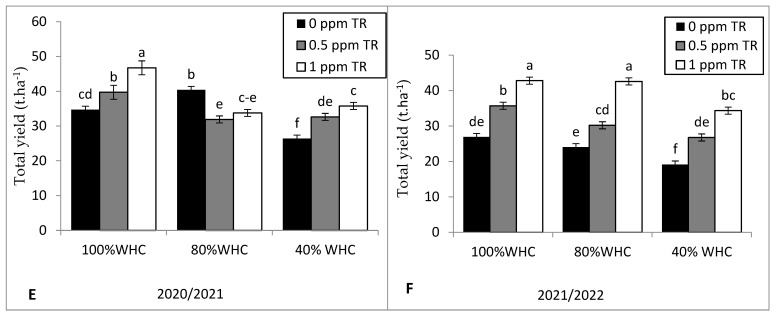
Effects of the interaction between water irrigation levels and foliar application with TR interaction on (**A**,**B**) total yield per plant (g) in 2020 and 2021, (**C**,**D**) early yield (kg·m^−2^) in 2020 and 2021, and (**E**,**F**) total yield (ton·hec^−1^) in 2020 and 2021. Vertical bars represent standard errors of the mean; in each bar, values followed by different letters differ significantly at *p* = 0.05 according to the LSD test.

Severe drought stress (40% WHC) significantly reduced the average fruit fresh weight (Figure 3A,B), number of fruits per plant (Figure 3C,D), total yield per plant (g) (Figure 4A,B), early yield (kg·m^−2^)(Figure 4C,D), and total yield (t ha^−1^) (Figure 4E,F) compared with recommended irrigation level (100% WHC). Triacontanol (1 ppm) applications increased average fruit fresh weight, number of fruits per plant, total yield per plant, early yield, and total fruit yield (ton·ha^−1^) in both years of study with control plants under normal and drought condition.

### 3.4. Fruit Quality Parameters

Severe drought stress (40% WHC) significantly decreased fruit length and fruit diameter when compared with moderate and normal irrigation (100% and 80% WHC) in both seasons (Table 5 and Table 6). The high level of TR application (1 ppm) recorded bigger and taller fruit in both seasons compared with control plants. Concerning the effect of interaction between water levels and TR application on fruit diameter and length, TR application at a rate of 1 ppm increased fruit diameter and fruit length under normal and moderate drought stress in both seasons.

Both drought levels, in both seasons, increased the content of TSS% and firmness in strawberry fruits compared with well-watered plants (Table 5 and Table 6). Both TR concentrations enhanced TSS content and firmness of the fruits under all water treatments.

**Figure 5:**
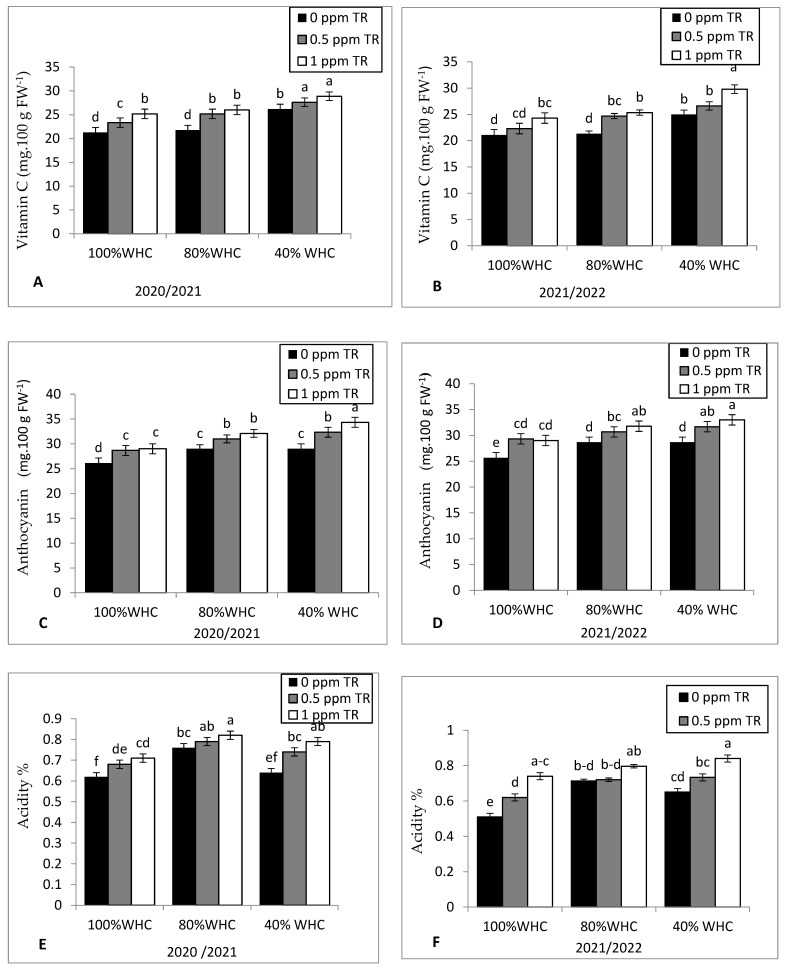
Effects of water irrigation levels and foliar application with TR interaction on (**A**,**B**) vitamin C during 2020 and 2021 seasons, respectively, (**C**,**D**) anthocyanin during 2020 and 2021 seasons, respectively, and (**E**,**F**) acidity during 2020 and 2021 seasons, respectively. Vertical bars represent standard errors of the mean; in each bar, values followed by different letters differ significantly at *p* = 0.05 according to the LSD test.

The same trend of results was observed in vitamin C (Figure 5A,B), anthocyanin content (Figure 5C,D), and acidity (Figure 5E,F) in both seasons. TR foliar treatments with either 0.5 or 1 ppm increased vitamin C content under severe drought stress in the first season. However, the high level of TR (1 ppm) recorded the highest vitamin C under severe drought stress in the second season. Both TR foliar treatments (0.5 and 1 ppm) increased anthocyanin content and acidity under both drought levels in the first season.

### 3.5. Macronutrients Content in Shoots

**Figure 6:**
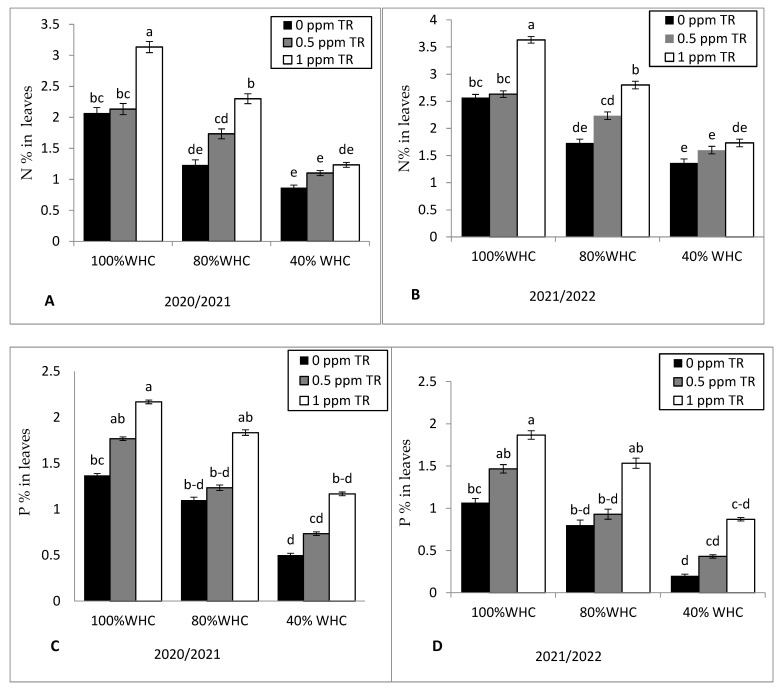

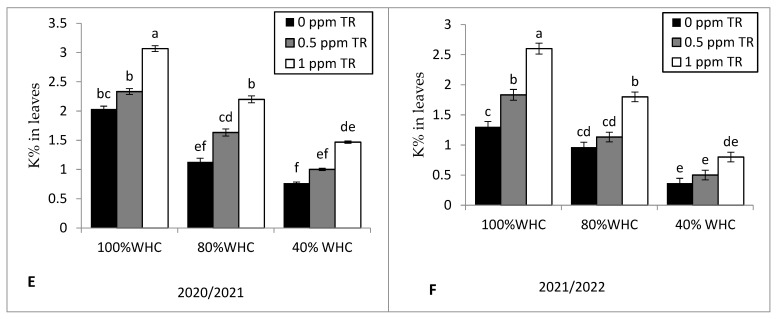
Effects of water irrigation levels and foliar application with TR interaction on (**A**,**B**) N% in leaves of strawberry in 2020 and 2021, (**C**,**D**) P% in leaves in 2020 and 2021, and (**E**,**F**) K% in leaves of strawberry in 2020 and 2021. Vertical bars represent standard errors of the mean; in each bar, values followed by different letters differ significantly at *p* = 0.05 according to the LSD test.

The data in Figure 5 reveal that moderate and severe water stress caused slight decreases in the values of the N% (Figure 6A,B), P% (Figure 6C,D), and K% (Figure 6E,F) compared to normal irrigation (100% WHC) in both seasons. TR application at 1 ppm increased N, P, and K contents of strawberry leaves under well-watered, moderate, and severe water condition. There was no significant difference between 0.5 ppm of TR and control treatment.

### 3.6. Antioxidant Enzymes and Hormones

**Figure 7:**
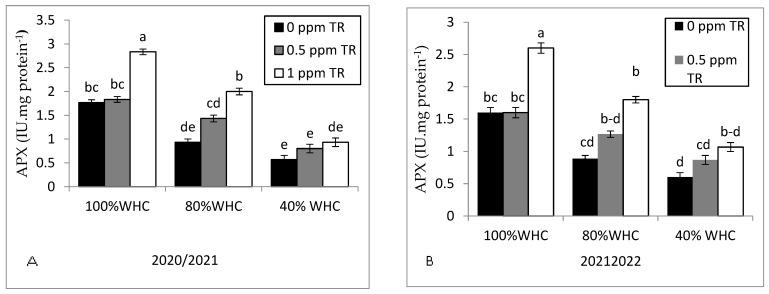

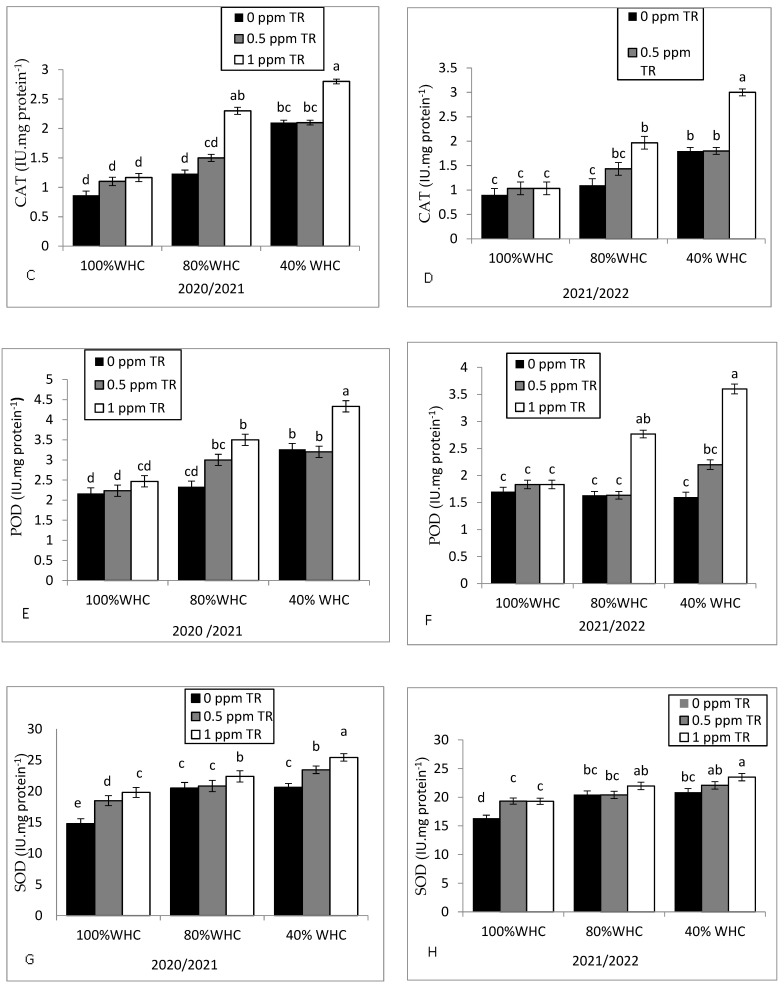

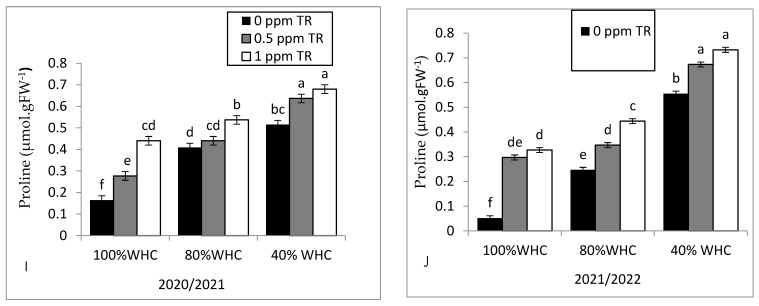
Effects of water irrigation levels and foliar application with TR interaction on (**A**,**B**) APX in leaves in 2020 and 2021, (**C**,**D**) CAT in leaves in 2020 and 2021, (**E**,**F**) POD in leaves in 2020 and 2021, (**G**,**H**) SOD in leaves in 2020 and 2021, and (**I**,**J**) proline in leaves in 2020 and 2021. Vertical bars represent standard errors of the mean; in each bar, values followed by different letters differ significantly at *p* = 0.05 according to the LSD test.

Severe drought stress (40% WHC) showed the lowest significant activity of APX (Figure 7A,B) compared with the rest of treatments in both seasons. However, severe drought treatment significantly increased CAT (Figure 7C,D), POD (Figure 7E,F), and SOD (Figure 7G,H) activities and proline (Figure 7I,J) compared to the well-watered condition. In contrast, foliar applications with TR at rates of 1 ppm increased the activity of CAT, POD, SOD, and APX enzymes and proline either under the well-watered condition or drought stress conditions. There was no difference between 0.5 ppm TR and control treatment.

### 3.7. Traits Interrelationship

**Figure 8:**
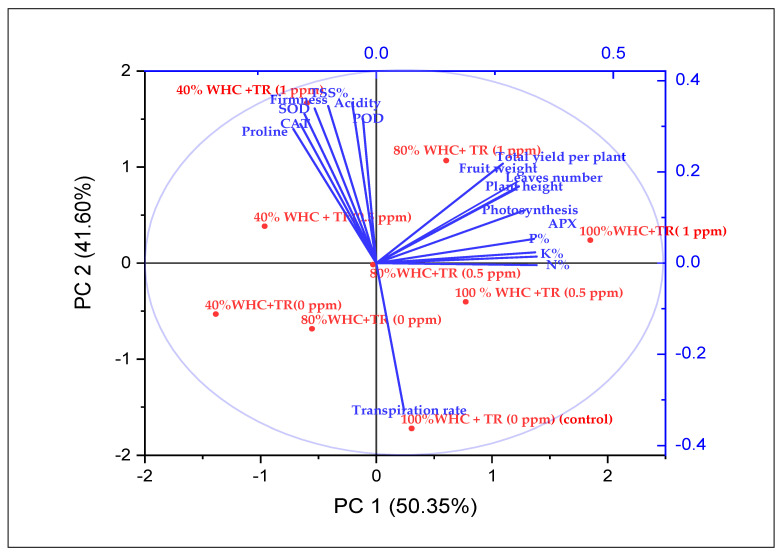
Biplot of the first two principal components for the morphological, yield, and physiochemical traits of strawberry plants. The morphological parameters comprised plant height and number of leaves per plant. The yield parameters included fruit weight and total yield. The fruit quality parameters included vitamin C, total soluble solids (TSS%), acidity, and firmness. The physiological traits included photosynthesis and transpiration rate. The physiochemical parameters comprised total nitrogen (N), phosphorus (P), potassium (K), superoxide dismutase (SOD), catalase (CAT), peroxidase (POD), and proline. Red circle symbols represent the different water regimes and TR treatments; 100% WHC + TR (0 ppm) control, 100% WHC + TR (0.5 ppm), 100% WHC + TR (1 ppm), 80% WHC + TR (0 ppm), 80% WHC + TR (0.5 ppm), 80% WHC + TR (1 ppm), 40% WHC + TR (0 ppm), 40% WHC + TR (0.5 ppm), 40%WHC + TR (1 ppm).

The association among evaluated morphological, yield, and physio-chemical traits of strawberry plants was estimated based on the analysis of principal components. Data were analysed using PCA in order to establish a relationship between water regime levels and foliar application with TR on plant growth and yield parameters. The score plots generated from PCA of sonicated and control strawberry plants are presented in Figure 8. The distribution of plant growth and yield parameters in space defined using the PCA dimensions is shown in Figure 8. The sum of principal components 1 and 2 (PC1 and PC2) accounted for 91.95% of variations among strawberry plants. PC1, the first component, contributed 50.35% of the total variation, and the second component accounted for 41.60% of the total variation. TR foliar treatments with either (0.5 or 1 ppm) under normal (100% WHC) and moderate (80% WHC) irrigation exhibited improved plant growth with higher plant height, number of leaves per plant, fruit weight, and total yield. In addition, they demonstrated increased photosynthesis and mineral content (nitrogen, phosphorus, and potassium) in leaves. In contrast, TR foliar treatments with either 0.5 or 1 ppm under severe drought stress (40% WHC) exhibited higher fruit quality, including vitamin C, firmness, TSS, and acidity. In addition, they displayed higher concentrations of leaf proline and antioxidant enzymes (SOD, POD, and CAT) due to oxidative injury under drought stress.

## 4. Discussion

In our study, drought stress induced suppression in plant growth parameters in strawberry plants. The decline in plant growth under water deficit stress (Table 1, Table 2, Table 3 and Table 4) is likely due to the detrimental osmotic influence of drought stress causing a decrease in photosynthetic pigments and disturbances of transpiration rate and water use efficiency [6,13,19]. The drought effects are motivate stomata closure, ionic balance disorder, and reduce photosynthesis, which decrease the plant growth and productivity [27,28,43]. Plant growth decreased due to water stress [44,45] because the strawberry plant is sensitive to drought stress and has a shallow root structure and high leaf area requiring a high quantity of water [46]. In our results, chlorophyll content decreased due to drought stress, which is in harmony with a previous study [47]. The role of TR in increasing plant growth under stress could be due to maintenance of water homeostasis, increased uptake of water and essential nutrients, and synthesis/accumulation of organic compounds [14]. The results obtained in this study were in harmony with the findings of Chartzoulakis et al. [48] who found that chlorophyll content was reduced with each decrease in soil moisture levels. Our results showed that foliar application of TR at both levels (0.5 and 1 ppm) significantly improved plant growth of strawberry plants under drought stress condition. The improvement in the plant growth could be due to the role of TR in enhancing the photosynthesis process by enhancing chlorophyll synthesis and increasing the number and size of chloroplasts [26,49]. This result is in agreement with previous works in some crops such as mung bean [50] and sunflower [51].

Our results showed that total yield and its component decreased due to water stress. Similar results were achieved from a study evaluating the impact of drought stress on strawberry yield [4,9].

In this study, application of TR increased the total yield and its components under normal and drought conditions. The role of TR in enhancing productivity might be due to the modulation in antioxidant activities [17] and regulation of photosynthetic genes [12]. Similar results were achieved from a study evaluating the impact of triacontanol on the yield of tomato [52,53]. They found that TR application improved the number of fruits, fruit weight, and total yield of tomato plants.

Drought stress reduced the nutritional content in leaves of strawberry plants, causing deficiencies in N%, P%, and K%, particularly under sever and moderate drought stress conditions. Our results are in agreement with Badawy et al. [54].

The results of this study supported our hypothesis that TR treatment enhanced the uptake of mineral content. In agreement with our study, previous work indicated that TR regulates different physiological and biochemical processes including the uptake and use efficiency of different mineral ions either under normal or stress conditions [55]. Moreover, TR plays an important role in water uptake, increasing cell division, cell elongation, and permeability of membranes [56]. TR can also enhance Ca^2+^, Mg^2+^, and K^+^ uptake [57].

Our results indicated that high drought stress increased the accumulation of anthocyanin, acidity, and TSS in fruits. Similar results were achieved from a study evaluating the impact of drought stress on the fruit quality of strawberry [9] and tomato [54]. Our results indicated that TR treatments improved the concentrations of vitamin C and anthocyanin under all water regime treatments. This result might be due to many reasons such as enhanced activation of metabolic activities including photosynthesis and enhanced enzyme activities that promote the plant growth [55,58]. In addition, it helps in the activation of the enzymes involved in vital physiological processes and carbohydrate metabolism [59]. The application of TR at a rate of 1 ppm improved macronutrient uptakes. Higher levels of macro nutrients in TR-treated plants could be related to the higher metabolic activity and increased dry matter production that enhanced water and nutrient uptake [60].

In our results, antioxidant enzymes (SOD, CAT, and POD) increased due to drought stress, which is in harmony with a previous study [60]. Our results in Figure 6 support our hypothesis that SOD, CAT, and APX are noticeably elevated in strawberry plants to enable them to tolerate drought stress. However, the strawberry plants failed to perform well under severe drought stress. Therefore, it was necessary to treat strawberry plants with TR to overcome the drought stress. Hence, foliar spraying with TR at a rate of 1 ppm noticeably enhanced growth parameters, yield traits, and physio-biochemical attributes of water deficit-stressed strawberry plants.

In a previous study [26] and our study (Figure 7I,J), TR foliar application led to markedly increased free proline under a severe water regime. Priming seed treatment with triacontanol has been found to enhance free proline in canola grown under saline conditions [61]. The accumulation of proline in stressed plants helps in reducing the osmotic potential of the plant cells that mitigate water stress [62]. The role of proline in reducing the harmful effect of drought stress in plants might be due to maintenance of membrane integrity and osmotic adjustment as well as enhancement of the antioxidant defense system [63,64].

TR application at a rate of 1 ppm recorded the highest significant increase in APX, CAT, POD, and SOD under well-watered and drought stress conditions (Figure 7). Our results are in agreement with Suman et al. [65] who found that activity of CAT, POD, and SOD enzymes was increased via TR application. Furthermore, TR controls the stress-related genes and up-regulates the genes involved in improved antioxidant enzymes [19]. TR also works as a good antioxidizing mediator [66] and reduces the collapse of lipid peroxidation of both non enzymatic and enzymatic reactions [67]. In parallel with our results, foliar application of TR enhanced the activity of POD in wheat [68]. In contrast to our findings, foliar application of TR did not change the SOD activity [21]. This difference with our result could be due to the different of plant type or the TR application method and concentration.

The interrelationship among the evaluated parameters (Figure 8) indicates that the yield parameters are positively associated with plant height, number of leaves, photosynthesis, and mineral content of leaves. We speculate that the high values of these photosynthesis rates are associated with the greater total yield and its contributing traits. In addition, proline showed a highly positive association with antioxidant activity including SOD, POD, CAT, and fruit quality (TSS%, acidity, and firmness). Furthermore, the proline content and fruit quality exhibited a highly negative association with transpiration rate.

## 5. Conclusions

**Figure 9:**
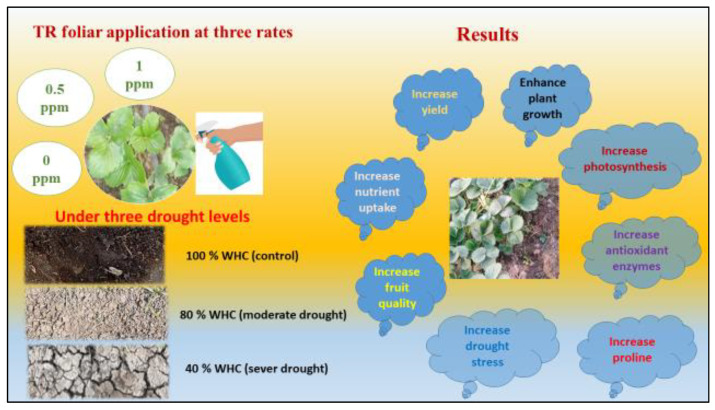
Graphical chart explains the effect of three levels of TR on physiological and biochemical response of strawberry plants under normal and drought stress conditions.

In this study, our resulted indicated that drought stress decreased the growth and yield of strawberry plants. However, drought stress increased the accumulation of anthocyanin, acidity, and TSS. Drought stress increased the activities of CAT, POD, and SOD enzymes, as well as proline content, while APX was decreased. Additionally, application of TR was effective in minimizing the harm of drought stress on plant growth and productivity of strawberry plants via increasing plant height, leaf area, plant fresh and dry weight, and total yield (Figure 9). In addition, TR application improved fruit quality including TSS, vitamin C, and anthocyanin. More molecular studies are required to understand the mechanism of TR in improving plants tolerance to abiotic stresses. Finally, supplementary applications of TR on strawberry plants are recommended to minimize the damage of drought stress, which could be useful for commercial production and the private sector.

## Figures and Tables

**Table 1:** Effect of the interaction between water irrigation levels and triacontanol (TR) foliar applications on growth parameters of strawberry plants in 2020/2021.

Irrigation (IR)	100% WHC	80% WHC	40% WHC	Mean	100% WHC	80% WHC	40% WHC	Mean
Treatments	Plant Height (cm)				Number of Leaves			
0 ppm TR	12.43 e	19.67 b	9.0 g	13.70 c	9.667 de	15.33 b	6.67 f	10.56 c
0.5 ppm TR	17.67 c	11.00 f	15.53 d	14.73 b	16.33 b	8.0 ef	11.0 cd	11.78 b
1 ppm TR	28.00 a	17.60 c	18.33 c	21.31 a	18.33 a	12.67 c	12.67 c	14.56 a
Mean	19.37 a	16.09 b	14.29 c		14.78 a	12.00 b	10.11 c	
LSD 0.05								
IR	0.7				1.0			
TR	0.73				1.1			
IR XTR	1.3				1. 9			
Treatments	Leaf Area (cm^2^)				Chlorophyll (SPAD) Reading			
0 ppm TR	44.00 d	56.33 b	31.67 f	44.00 c	30.33 b	29.67 bc	27.3 d	29.11 b
0.5 ppm TR	58.00 b	40.67 e	44.33 d	47.67 b	31.33 b	30.67 b	28.0 cd	30.00 b
1 ppm TR	62.00 a	50.67 c	50.33 c	54.33 a	36 a	30.67 b	29.33 bcd	31.89 a
Mean	54.67 a	49.22 b	42.11 c		32.44 a	30.33 b	28.22 c	
LSD 0.05								
IR	1.0				1.16			
TR	1.2				1.2			
IR XTR	2.2				2.02			

Values followed by the same letter are not significant according to the LSD test (*p* ≤ 0.05%).

**Table 2:** Effect of the interaction between water irrigation levels and triacontanol (TR) foliar applications on growth parameters of strawberry plants in 2021/2022.

Irrigation (IR)	100% WHC	80% WHC	40% WHC	Mean	100% WHC	80% WHC	40% WHC	Mean
Treatments	Plant Height (cm)				Number of Leaves			
0 ppm TR	11.77 d	10.3 d	8.3 e	10.14 c	9.333 d	7.333 e	6.333 e	7.667 c
0.5 ppm TR	17.33 b	14.20 c	11.1 d	14.0 b	14.67 b	11.67 c	10.33 d	12.22 b
1 ppm TR	20.33 a	17.30 b	15.20 c	17.61 a	17.33 a	13.67 b	12.00 c	14.33 a
Mean	16.48 a	13.94 b	11.57 c		13.78 a	10.89 b	9.556 c	
LSD.005								
IR	1.0				0.73			
TR	1.02				1.0			
IR XTR	1.7				1.2			
Treatments	Leaf Area (cm^2^)				Chlorophyll (SPAD) Reading			
0 ppm TR	42.0 d	36.0 ef	32.67 f	36.89 c	31.33 b–d	29.67 d	27.0 e	29.33 b
0.5 ppm TR	51.3 b	41.0 d	37.0 e	43.11 b	32.67 b	31. 7 b–d	26.67 e	30.33 b
1 ppm TR	60.0 a	53.0 b	46.0 c	53.00 a	35.67 a	32.0 bc	30.0 cd	32.56 a
Mean	51.11 a	43.3 b	38.56 c		33.22 a	31.11 b	27.89 c	
LSD.005								
IR	1.942				1.239			
TR	2.0				1.239			
IR XTR	3.363				2.146			

Values followed by the same letter are not significant according to the LSD test (*p* ≤ 0.05%).

**Table 3:** Effect of the interaction between water irrigation levels and triacontanol (TR) foliar applications on growth parameters of strawberry plants in 2020/2021.

Irrigation (IR)	100% WHC	80% WHC	40% WHC	Mean	100% WHC	80% WHC	40% WHC	Mean
Treatments	Shoot Fresh Weight (g)				Shoot Dry Weight (g)			
0 ppm TR	35.0 e	27.0 f	22.33 f	28.11 c	9.0 cd	8.0 de	6.667 e	7.889 b
0.5 ppm TR	48.0 bc	42.3 d	33.67 e	41.33 b	13.07 a	11.1 b	10.67 bc	11.61 a
1 ppm TR	51.3 ab	53.3 a	45.33 cd	50.00 a	13.07 a	11.67 ab	11.13 b	11.96 a
Mean	44.8 a	40.89 b	33.78 c		11.71 a	10.26 b	9.489 b	
LSD.005								
IR	0.64				1.0			
TR	2.792				1.1			
IR XTR	4.836				1.94			
Treatments	Root Fresh Weight (g)				Root Dry Weight (g)			
0 ppm TR	7.00 de	6.0 e	5.33 e	6.11 c	1.90 d	1.033 e	0.96 f	1.30 c
0.5 ppm TR	10.67 b	9.0 bc	8.33 cd	9.33 b	3.00 b	2.533 c	2.23 cd	2.589 b
1 ppm TR	13.00 a	10.47 b	10.10 b	11.19 a	3.900 a	3.167 b	3.133 b	3.40 a
Mean	10.22 a	8.489 b	7.92 b		2.933 a	2.244 b	2.111 b	
LSD.005								
IR	1.001				0.20			
TR	1.0				0.20			
IR XTR	1.73				0.36			

Values followed by the same letter are not significant according to the LSD test (*p* ≤ 0.05%).

**Table 4:** Effect of the interaction between water irrigation levels and triacontanol (TR) foliar applications on growth parameters of strawberry plants in 2021/2022.

Irrigation (IR)	100% WHC	80% WHC	40% WHC	Mean	100% WHC	80% WHC	40% WHC	Mean
Treatments	Shoot Fresh Weight (g)				Shoot Dry Weight (g)			
0 ppm TR	36.67 c	53.33 a	23.33 e	37.78 b	9.333 d	12.33 abc	7.00 e	9.556 c
0.5 ppm TR	47.00 b	29.00 d	34.67 c	36.89 b	12.73 ab	8.333 de	11.10 c	10.72 b
1 ppm TR	55.00 a	44.67 b	46.67 b	48.78 a	13.57 a	11.77 bc	11.47 bc	12.27 a
Mean	46.22 a	42.33 b	34.89 c		11.88 a	10.81 b	9.856 c	
LSD.005								
IR	1.8				0.8			
TR	2.0				1.0			
IR XTR	3.2				1.5			
Treatments	Root Fresh Weight (g)				Root Dry Weight (g)			
0 ppm TR	7.667 de	11.47 a	5.667 f	8.267 b	1.713 e	3.167 b	0.92 f	1.934 c
0.5 ppm TR	10.00 bc	6.667 ef	9.0 cd	8.556 b	3.067 b	1.043 f	2.057 d	2.056 b
1 ppm TR	12.13 a	9.467 bc	10.80 ab	10.80 a	3.767 a	2.567 c	3.017 b	3.117 a
Mean	9.933 a	9.200 ab	8.489 b		2.849 a	2.259 b	1.999 c	
LSD.005								
IR	0.83				0.1			
TR	1.0				0.1			
IR XTR	1.4				0.2			

Values followed by the same letter are not significant according to the LSD test (*p* ≤ 0.05%).

**Table 5:** Effect of water irrigation (IR) levels, triacontanol (TR) foliar applications, and their interactions on fruit quality parameters of strawberry plant in 2020/2021.

Irrigation (IR)	100% WHC	80% WHC	40% WHC	Mean	100% WHC	80% WHC	40% WHC	Mean
Treatments	Total Soluble Acids %				Fruit Diameter (cm)			
0 ppm TR	4.4 d	5.6 bc	6.0 bc	5.4 b	2.4 c–e	2.07 de	1.5 e	1.97 b
0.5 ppm TR	5.0 cd	5.8 bc	6.43 b	5.7 b	3.3 bc	2.7 cd	1.6 e	2.5 b
1 ppm TR	5.67 bc	6.80 b	8.03 a	6.8 a	4.4 a	3.9 ab	3.1 bc	3.8 a
Mean	5.033 c	6.17 b	6.82 a		3.4 a	2.8 a	2.06 b	
LSD.005								
IR	0.7				0.5			
TR	0.7				0.6			
IR XTR	1.2				0.9			
Treatments	Firmness (kg·m^−2^)				Fruit Length (cm)			
0 ppm TR	0.09 e	0.25 bc	0.22 b–d	0.19 b	3.5 d–f	3.3 ef	2.7 f	3.2 c
0.5 ppm TR	0.15 de	0.19 b–d	0.29 ab	0.21 ab	4.6 bc	3.9 c–e	2.9 f	3.8 b
1 ppm TR	0.19 c–e	0.22 b–d	0.38 a	0.26 a	5.8 a	5.2 ab	4.4 b–d	5.2 a
Mean	0.146 c	0.22 b	0.29 a		4.6 a	4.2 a	3.4 b	
LSD.005								
IR	0.05				0.58			
TR	0.06				0.6			
IR XTR	0.09				1.1			

Values followed by the same letter are not significant according to the LSD test (*p* ≤ 0.05%).

**Table 6:** Effect of water irrigation level and triacontanol (TR) foliar applications on fruit quality traits of strawberry plant during 2021/2022.

Irrigation (IR)	100% WHC	80% WHC	40% WH	Mean	100% WHC	80% WHC	40% WHC	Mean
Treatments	Total Soluble Acids %				Fruit Diameter (cm)			
0 ppm TR	4.6 d	5.3 cd	5.3 cd	5.04 c	3.2 c–e	3.0 de	2.4 e	2.8 c
0.5 ppm TR	5.03 d	5.9 bc	6.6 ab	5.9 b	4.3 ab	4.0 b–d	2.6 e	3.5 b
1 ppm TR	5.3 d	6.6 ab	7.3	6.4 a	4.9 a	5.0 a	4.1 a–c	4.6 a
Mean	4.9 c	5.9 b	6.4 a		4.1 a	3.8 a	3.1 b	
LSD.005								
IR	0.4				0.5			
TR	0.43				0.6			
IR XTR	0.74				1.0			
Treatments	Firmness (kg·m^−2^)				Fruit Length (cm)			
0 ppm TR	0.13 e	0.2 cd	0.23 a–d	0.1878 b	3.3 c–e	2.9 de	2.4 e	3.0 b
0.5 ppm TR	0.18 de	0.22 b–d	0.24 a–c	0.21 b	4.2 bc	3.6 cd	2.50 e	3.4 b
1 ppm TR	0.19 cd	0.270 ab	0.29 a	0.25 a	5.34 a	4.8 ab	4.0 bc	4.7 a
Mean	0.17 b	0.23 a	0.25 a		4.28 a	3.8 a	2.9 b	
LSD.005								
IR	0.03				0.55			
TR	0.03				0.6			
IR XTR	0.06				0.9			

Values followed by the same letter are not significant according to the LSD test (*p* ≤ 0.05%).

## Data Availability

Data is contained within the article.
